# Fiber Optic Fabry-Perot Interferometer Pressure Sensors for Oil Well

**DOI:** 10.3390/s25206297

**Published:** 2025-10-11

**Authors:** Zijia Liu, Jin Cheng, Jinheng Li, Junming Li, Longjiang Zhao, Zhiwei Zheng, Peizhe Huang, Hao Li

**Affiliations:** 1 School of Applied Science, Beijing Information Science and Technology University, Beijing 102206, China; l906280177@163.com (Z.L.); li@bistu.edu.cn (J.L.);; 2 Beijing Key Laboratory for Sensor, Ministry of Education Key Laboratory for Modern Measurement and Control Technology, Beijing 102206, China; 3 School of Engineering, Qufu Normal University, Rizhao 276800, China; 4 Beijing SKC Acoustic Technology Co., Ltd., Beijing 100015, China

**Keywords:** fiber optic Fabry–Perot sensor, high temperature and high-pressure environment, temperature-pressure decoupling, well monitoring

## Abstract

In oil well environments, pressure sensors are often challenged by electromagnetic interference, temperature drift, and corrosive fluids, which reduce their stability and service life. To improve long-term reliability under these conditions, we developed a fiber optic Fabry–Perot (FP) cavity pressure sensor that employs an Inconel 718 diaphragm to provide both high mechanical strength and corrosion resistance. An integrated fiber Bragg grating (FBG) was included to monitor temperature simultaneously, allowing temperature–pressure cross-sensitivity to be decoupled. The sensor was fabricated and tested over a temperature range of 20–100 °C and a pressure range of 0–60 MPa. Experimental characterization showed that the FP cavity length shifted linearly with pressure, with a sensitivity of 377 nm/MPa, while the FBG demonstrated a temperature sensitivity of 0.012 nm/°C. After temperature compensation, the overall pressure measurement accuracy reached 0.5% of the full operating pressure range (0–60 MPa). These results confirm that the combined FP–FBG sensing approach maintained stable performance in harsh downhole conditions, making it suitable for pressure monitoring in shallow and medium-depth reservoirs. The proposed design offers a practical route to extend the operational lifetime of optical sensors in oilfield applications.

## 1. Introduction

In oil exploration and production, real-time monitoring of downhole pressure and temperature is critical for operational safety, production optimization, and extending the equipment lifetime. In China, conventional oil reserves are mainly distributed in shallow (<2000 m) and medium-depth (2000–3500 m) formations, accounting for about 54% and 26% of the national total, respectively [1]. At depths around 3000 m, the formation temperature typically reaches 80 °C. Consequently, pressure sensors for such reservoirs must operate reliably within a pressure range of 0–60 MPa and withstand elevated temperatures of at least 80 °C [2,3].

Conventional electrical pressure sensors encounter serious challenges under these conditions. They are vulnerable to electromagnetic interference, thermal drift, mechanical fatigue, and corrosion, which undermine their long-term stability [4,5]. The coexistence of high temperature and pressure in deep wells further produces thermo-mechanical coupling effects, amplifying measurement errors. In addition, transmission delays in electrical systems hinder timely data acquisition and processing, limiting their effectiveness in field applications [6,7].

With the rapid development of fiber optic sensing technology, optical pressure sensors have attracted attention for downhole use due to their compact size, fast response, immunity to electromagnetic interference, and high resistance to thermal and chemical degradation [8,9]. Among these, fiber optic Fabry–Perot (FP) cavity pressure sensors stand out for their simple configuration and high sensitivity [10,11]. However, most domestic designs rely on epoxy resin encapsulation [12,13]. Under high-temperature wellbore conditions, epoxy encapsulants deform, altering the FP cavity length and degrading both accuracy and stability. Thus, conventional encapsulation strategies fail to ensure reliable long-term performance [14,15].

To address this issue, temperature–pressure decoupling is essential. In this work, fiber Bragg gratings (FBGs) were introduced as temperature references to enable the compensation of thermally induced pressure errors. A fiber optic FP cavity pressure sensor with metal diaphragm encapsulation was developed, combined with an FBG-assisted temperature compensation scheme. The sensor was tested over 20–100 °C and 0–60 MPa, demonstrating effective decoupling of temperature and pressure responses. The experimental results indicate a measurement accuracy of 0.5% of the full operating pressure range (0–60 MPa), corresponding to ±0.3 MPa, and good thermal stability, highlighting its suitability for practical downhole applications.

## 2. Materials and Methods

### 2.1. Principles

**Figure 1:**
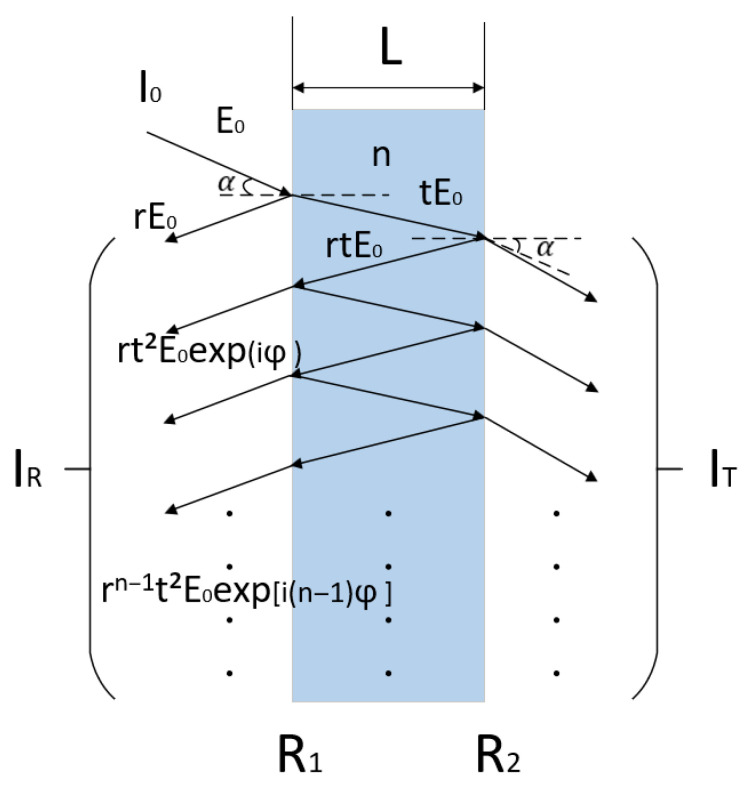
Multi-beam interferometric optical model.

Figure 1 shows the multi-beam interference optical model in a Fabry–Perot (FP) interferometer, where L is the FP cavity length, λ is the incident light wavelength, n is the refractive index of the medium between the planes, and the path difference between adjacent reflected light beams satisfies the following:

(1) $$\begin{array}{c} 2nLcos\alpha =m\lambda \left(m=1,2,3,\ldots \right) \end{array}$$

The phase difference satisfies:

(2) $$\varphi =\frac{4\pi }{\lambda }nLcos\alpha$$

The reflectance of the two coated surfaces is $R_{1}$ and $R_{2}$, assuming that the reflectance $R_{1}=R_{2}=r$ [16], and the transmittance is t. According to Figure 1, the amplitude of the reflected light $E_{1}=rE_{0}$ after reflection by surface $R_{1}$. After n reflections by $R^{2}$, the total amplitude of the reflected light spectrum $E_{r}$ is:

(3) $$E_{r}=\sum_{n=0}^{\infty }En=\sum_{n=0}^{\infty }\{r^{n-1}t^{2}E_{0}\exp\left[i\left(n-1\right)\varphi \right]\}=\left[r+\frac{t^{2}r\exp\left(i\varphi \right)}{1-r^{2}\exp\left(i\varphi \right)}\right]E_{0}$$

Total reflected light intensity is:

(4) $$I_{R}=E_{r}E_{r}^{*}=\frac{2R\left(1-cos\varphi \right)}{1+R^{2}-2Rcos\varphi }$$

Ignoring absorption loss, when $R_{1}\neq R_{2}$, we have:

(5) $$I_{R}=ErE_{r}^{*}=I_{0}\frac{R_{1}+R_{2}-2\sqrt{R_{1}R_{2}}cos\varphi }{1+R_{1}R_{2}-2\sqrt{R_{1}R_{2}}cos\varphi }$$

According to the theory of minimal perturbation [17], the relationship between pressure and cavity length is shown in the following equation:

(6) $$∆d=\frac{3}{16}\frac{\left(1-\mu^{2}\right)r^{4}}{Eh^{3}}∆p$$

In the equation, the thickness of the membrane is h; the change in cavity length is ∆d; the change in membrane pressure is ∆p; the effective radius of the membrane is r; Poisson’s ratio of the sensitive membrane is μ; and Young’s modulus is E. By solving for the cavity length, the pressure value can be obtained. Here, the cavity length is demodulated using the wavelength corresponding to the interference peak in the interference spectrum [18]. When the light intensity $I_{R}$ is at its peak, the relationship between the wavelengths $\lambda_{m}$ and $\lambda_{m+n}$ at the mth and m+nth peak points of the interference fringes and the cavity length is as follows:

(7) $$L=\left(\frac{m}{2}+\frac{1}{4}\right)\lambda_{m}$$

(8) $$L=\left(\frac{m+n}{2}+\frac{1}{4}\right)\times \lambda_{m+n}$$

The expression for the cavity length is obtained by the following association:

(9) $$L=\frac{n}{2}\times \frac{\lambda_{m+n}\lambda_{m}}{\lambda_{m}-\lambda_{m+n}}$$

The wavelength information of neighboring interference peaks is extracted using the bimodal method to calculate the cavity length [19,20], and the amount of cavity length $L=L_{0}+∆d$. The value of the applied pressure can be obtained by calibrating the initial cavity length value $L_{0}$ and the demodulated cavity length value L after pressure application [21].

### 2.2. Analysis of Temperature–Pressure Coupling Effect

Fiber optic Fabry–Perot (FP) sensors are inevitably affected by the combination of ambient temperature and pressure in oil-well-oriented applications [22]. Temperature changes cause thermal expansion of the high-temperature adhesive and the sensing structure, as well as deformation of the metal diaphragm and the optical fiber, while the boundary pressure acting on the diaphragm causes it to undergo mechanical deformation, and these factors together affect the effective length of the FP chamber, which then changes the position and shape of the interference spectra, resulting in the typical temperature–pressure coupling effect [23,24].

In this study, the mathematical expression for the cavity length under temperature–pressure coupling is established based on the constructed structural model of the diaphragm-type fiber FP cavity. Let the initial temperature be *T*_0_ = 20 °C and the initial pressure be *P*_0_ = 0 MPa, and the change in cavity length can be described by the following equation:

(10) $$L=\left(L_{1}-L_{2}-L_{3}\right)+\left[\alpha_{1}\left(L_{1}-L_{2}\right)-\alpha_{2}L_{3}\right]\left(T-T_{0}\right)-Y_{P}\left(P-P_{0}\right)$$

**Figure 2:**
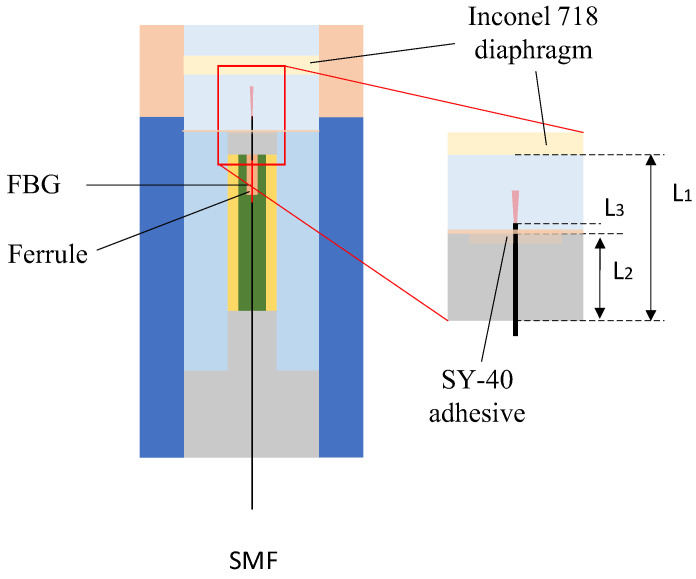
Schematic diagram of temperature–pressure coupling structure.

In Figure 2, $L_{1}$, $L_{2}$, $L_{3}$ represent the effective length between the diaphragm and the connector, the length between the fiber optic holder and the connector, and the effective length of the fiber optic ferrule; $\alpha_{1}$ and $\alpha_{2}$ are the coefficients of thermal expansion of the Inconel 718 material and the fiber optic fiber, $Y_{P}$ is the pressure sensitivity of the diaphragm, and T, P are the ambient temperatures andpressures of the boundary area. The diaphragm is fabricated from Inconel 718 alloy, which offers high yield strength, excellent fatigue resistance, and good corrosion resistance even at elevated temperatures. These properties make it particularly suitable for harsh environments such as oil wells [25].

To study the effect of temperature–pressure coupling on interference spectra, the parameters are set as follows: $L_{1}=5\;mm$, $L_{2}=4.5\;mm$, $L_{3}=0.25\;mm$, $\alpha_{1}=13.3\times 10^{-6}/{}^{\circ}C$, $\alpha_{2}=5.5\times 10^{-7}/{}^{\circ}C$, $Y_{P}=350\;nm/MPa$, and let $I_{0}=1\;W$.

## 3. Simulation Analysis and Sensor Design

### 3.1. Effects of Temperature and Pressure on the Sensor Spectra

**Figure 3:**
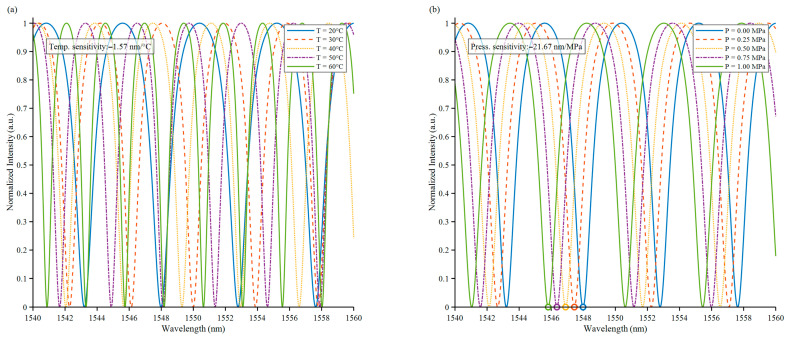
Investigating the effects of pressure and temperature changes on interference spectra. (**a**) Simulation of interference spectrum at different temperatures. (**b**) Simulation of interference spectrum under different pressures.

The sensor’s response to external temperature and pressure changes was characterized by numerical simulations. Under a constant pressure of 0 MPa, the temperature was increased from T = 20 °C to 60 °C in 10 °C increments, as shown in Figure 3a As the temperature increased, the interference spectrum shifted overall. Thermal expansion caused the FP cavity length to increase, resulting in an increase in the phase difference and a shift in the overall spectrum.

The pressure is increased from 0 MPa to 5 MPa with 5 MPa increment each time at a fixed temperature of T = 20 °C. Figure 3b shows the variation in the interference spectrum with pressure, the diaphragm undergoes a concave deformation under external pressure, and the cavity length decreases with the increase in pressure.

### 3.2. Temperature–Pressure Coupling Effect Simulation and Decoupling Strategy

**Figure 4:**
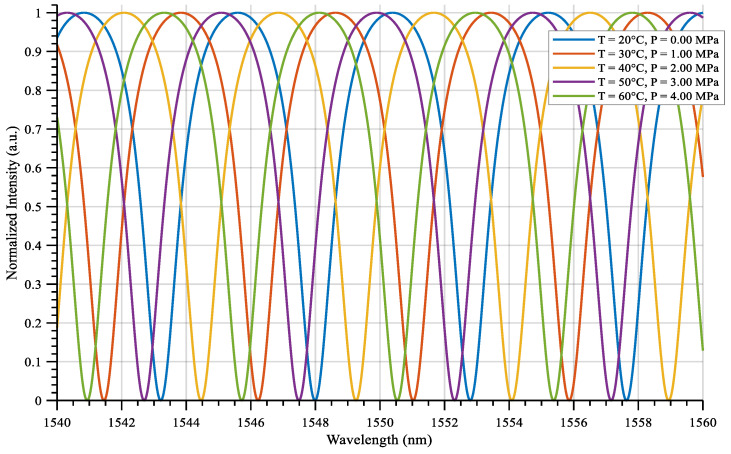
Simulation of temperature–pressure simultaneous interference spectrum.

When the pressure is measured in the actual oil well, the temperature and pressure affect the cavity length together under the simultaneous change in the two parameters, and the drift of the interference spectrum is no longer solely controllable. An example of the simulation results are shown in Figure 4, demonstrating when the pressure is varied from 0 to 4 MPa and the temperature is varied from 20 to 60 °C in steps.

In particular, the cavity length is 249,430 nm when the pressure is 2 MPa and the temperature is increased to 40 °C, while the cavity length is 249,300 nm when the same pressure is applied but the initial temperature is maintained at 20 °C, which is a difference of 130 nm. If the cavity length is interpreted as pressure detuned only by using the pressure sensitivity of the diaphragm, the error is about 0.37 MPa. This suggests that the uncoupled temperature effect can significantly interfere with the accuracy of the pressure measurement. This shows that without decoupling, temperature significantly interferes with the pressure measurement accuracy, which is the deviation caused by the temperature–pressure coupling effect [26,27].

When measuring pressure under the combined influence of temperature and pressure, it is necessary to decouple the temperature as follows: Record the initial cavity length $L_{0}$ of the sensor, corresponding to the initial pressure $P_{0}$ and initial temperature $T_{0}$. The temperature sensitivity of the cavity length is denoted as $Y_{T}$, the pressure sensitivity as $Y_{P}$, and the FBG temperature sensitivity as $Y_{FBG}$. When the temperature changes by ∆T, the change in cavity length is $∆L\left(T\right)$. When the pressure changes by ∆P, the change in cavity length is $∆L\left(P\right)$. Therefore, when both pressure and temperature act simultaneously, the value of the sensor’s cavity length L is

(11) $$L=L_{0}+∆L\left(T\right)+∆L\left(P\right)=L_{0}+Y_{T}\cdot ∆T+Y_{P}\cdot ∆P$$

Calibrate the temperature sensitivity $Y_{FBG}$ of the FBG under static pressure and the sensitivity $Y_{T}$ of the cavity length to temperature under static pressure. The current temperature is calculated from the spectral data of the FBG. Based on the current temperature and the calibrated temperature cavity length sensitivity, the cavity length change caused by temperature is calculated. The remaining cavity length change is the cavity length change caused by pressure.

### 3.3. Sensor Fabrication and Assembly

**Figure 5:**
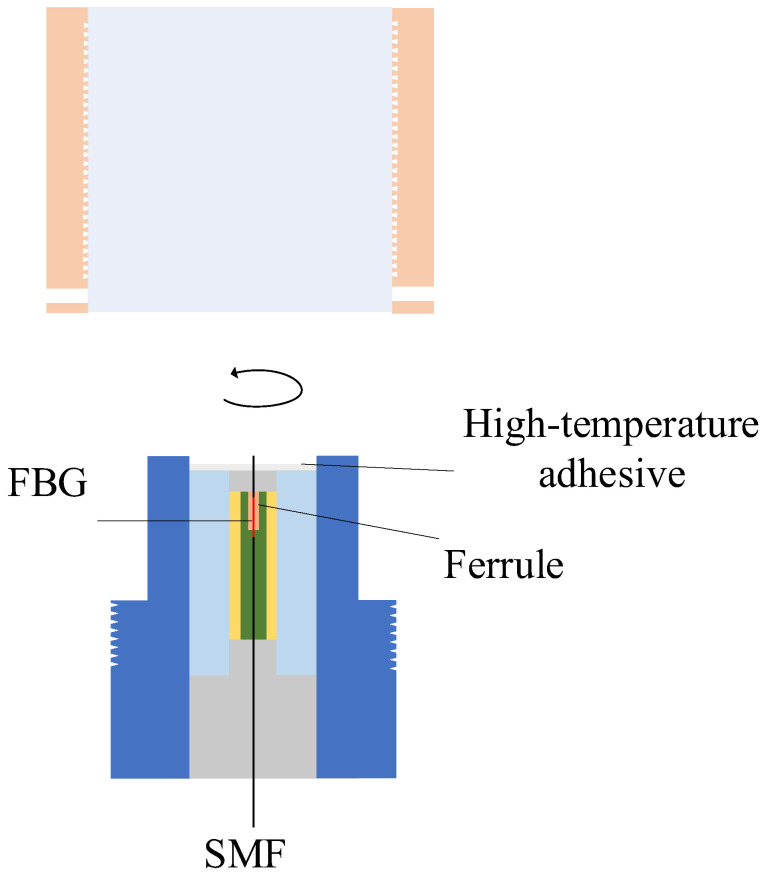
Fiber optic probe model. SMF: processed single-mode optical fiber. FBG: fiber Bragg grating. High-temperature adhesive: SY-40 adhesive, produced by the Beijing Institute of Aeronautical Materials, is supplied in two components. It has an operating temperature range of –55 °C to 200 °C, with a shear strength exceeding 16 MPa at room temperature and remaining above 2 MPa at 200 °C. Ferrule: A metal ferrule with an inner diameter slightly larger than the diameter of the optical fiber.

To experimentally validate the proposed method for sensor fabrication, the following steps were first performed during the preprocessing stage: using fiber optic cutters, wire strippers, and a spectrometer, the fiber optic cables with FBG were subjected to end-face processing and reflectance testing, and those with qualified reflectance performance were selected for use. As shown in Figure 5, the qualified optical fibers are inserted into the optical fiber ferrule, and SY-40 high-temperature adhesive is used for bonding and fixation. To ensure that the FBG region is not affected by stress, it remains in a free segment state within the ferrule.

**Figure 6:**
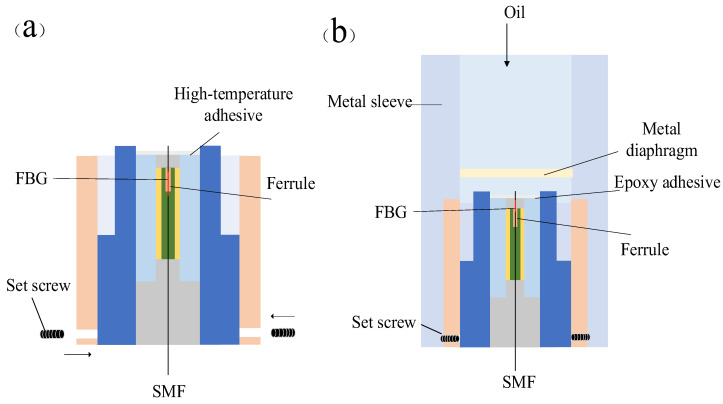
Fiber optic probe assembly. (**a**) Probe fixed with a clamp and set screw. (**b**) Schematic diagram of FP cavity assembly. Oil: oil enters the metal diaphragm through the top opening. Metal diaphragm: a pressure-resistant metal sensitive diaphragm made of Inconel 718. Metal sleeve: a metal outer shell made of Inconel 718 that protects the internal sensitive sensor components. Set screw: a metal set screw that is screwed into the sensor from all sides to secure the sensor probe.

The module is then assembled by screwing the probe structure into the central fixture. The fixture has openings around its perimeter. By tightening the M3 set screws to press down on the fiber optic probe, the position of the probe is secured, ensuring its stability, as shown in Figure 6a. The cavity assembly consists of a connector, a pressure membrane cylinder, and an integrated metal diaphragm. The diaphragm is made of Inconel 718 nickel-based alloy, with a thickness of approximately 1.6 mm, offering excellent mechanical strength and corrosion resistance. Since the visibility of interference fringes highly depends on the initial cavity length, to ensure the quality of the reflection spectrum, the interference pattern is monitored in real-time using a spectrometer during assembly, and the thread insertion depth is adjusted to obtain the optimal initial cavity length.

**Figure 7:**
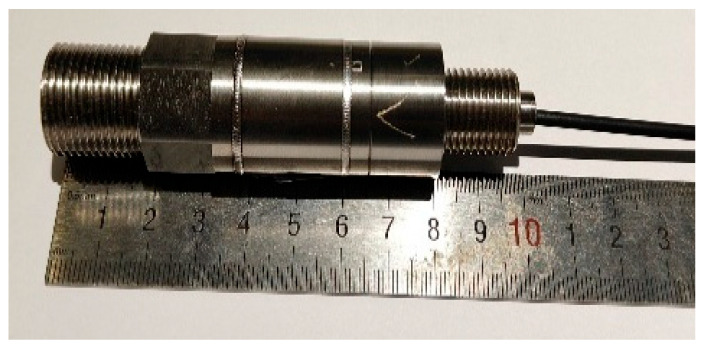
Overall diagram of the sensor. Each pure metal shell module is assembled and laser welded, with a length of 10 cm.

Finally, complete the module fixation and encapsulation protection as shown in Figure 6b. Insert a sleeve into the end of the optical fiber to protect the bare fiber; then tighten the connector. Fill the tail end and fiber exit with high-temperature glue to seal and protect the entire sensor structure. The final sensor physical diagram is shown in Figure 7.

### 3.4. Experimental Platform Configuration

**Figure 8:**
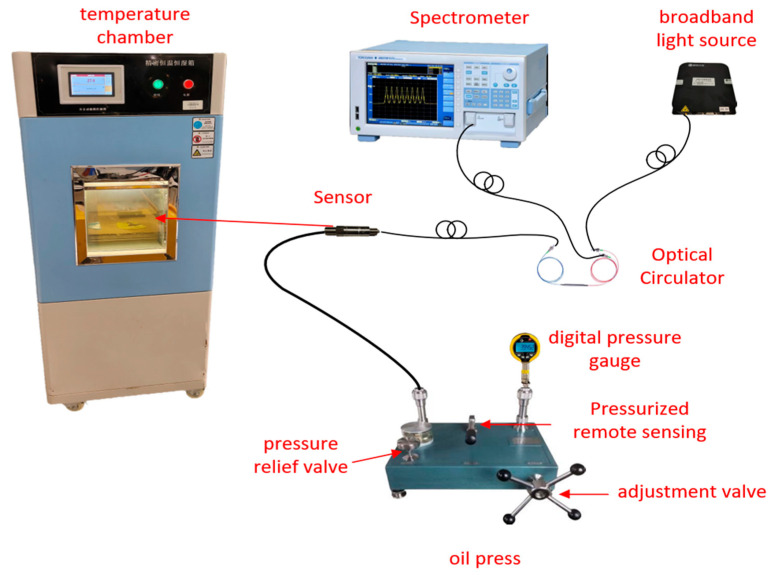
Experimental platform.

To verify the stability and measurement performance of the designed fiber optic FP cavity pressure sensor under high-pressure and high-temperature conditions, a complete experimental testing system was established, as shown in Figure 8. Light emitted by the ASE broadband light source passes through a fiber optic circulator and enters the pressure sensing unit. The interference signal is returned through the circulator to the spectrometer for real-time acquisition. The hydraulic pressure gauge delivers hydraulic pressure through a pipeline to the metal pressure diaphragm of the sensor, causing deformation of the diaphragm. The sensor is placed in a temperature-controlled chamber to control the temperature variable. This test system uses a pressure gauge with a range of 0–70 MPa and an accuracy of 0.05% FS; the temperature-controlled chamber provides a temperature regulation range of −40 °C to 120 °C with a resolution of 0.1 °C, and the chamber temperature is displayed in real time on the panel. Signal demodulation employs the double-peak method within the spectral peak tracking method to stably demodulate the interference fringes, thereby inversely deriving the dynamic curve of the FP cavity length as a function of pressure in real time [20].

## 4. Experimental Results and Analysis

### 4.1. Sensor Calibration

To verify the decoupling pressure measurement capability and accuracy of the proposed scheme, data calibration was performed on the pressure sensitivity $Y_{P}$, temperature sensitivity $Y_{T}$, and FBG temperature sensitivity $Y_{FBG}$ prior to the experiment. The calibration of $Y_{P}$ was performed using an equidistant method within the range of 0–60 MPa, with pressure data collected at intervals of 5 MPa. For the calibration of $Y_{FBG}$, an equidistant method was used within the temperature range of 20–80 °C, with data collected at intervals of 10 °C. To more accurately analyze the output response characteristics of the sensor, the pressure experiment was repeated three times, with the data shown in Table 1.

**Figure 9:**
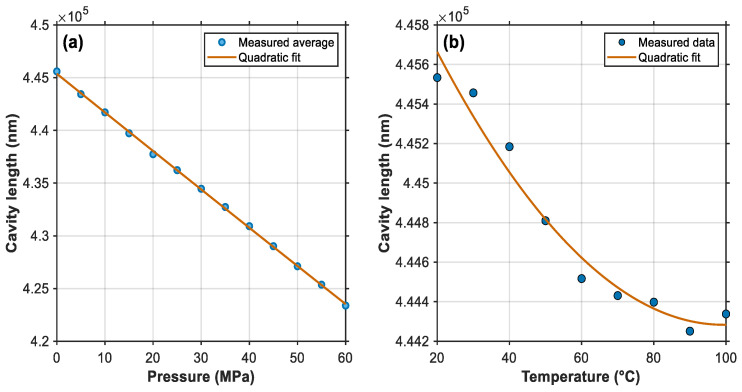
Cavity length fitting curve. (**a**) Pressure sensitivity fitting curve. (**b**) Fitted curve of temperature vs. cavity length sensitivity.

The arithmetic mean of the calculated data is used as the reference point. Processing the data yields the relationship between pressure and cavity length at a fixed temperature. The relationship curve between the two is shown in Figure 9a.

The curve fitting formula is as follows:

(12) L=445607.22−377.43⋅P

From the above equation, we can see that the pressure sensitivity $Y_{P}$ of the sensor is 377 nm/MPa. Similarly, keeping the pressure at 0 MPa, the temperature sensitivity $Y_{FBG}$ of the FBG is calibrated, and the relationship between temperature and center wavelength is:

(13) λ=1530.61069+0.01229∗T

The sensitivity $Y_{FBG}$ of the FBG is calculated to be 0.012 nm/°C. Since the linearity of the temperature effect on the cavity length is relatively low, the calibration of $Y_{T}$ is performed using a polynomial fitting method. The fitting curve of temperature and cavity length is shown in Figure 9b. The corresponding formula is as follows:

(14) $$L=0.262263*T^{2}-49.666869*T+446645.501476$$

(15) $$Y_{T}=\frac{dL}{dT}=0.524525*T-49.666869$$

### 4.2. Decoupling Capability Verification

After determining the calibrated sensitivities $Y_{T}$, $Y_{P}$, and $Y_{FBG}$, we evaluated the sensor performance using the experimental setup. Tests were carried out at temperatures of 20 °C, 30 °C, 50 °C, and 60 °C, with applied pressures of 15 MPa, 30 MPa, and 45 MPa. To exclude the effect of the sensor’s intrinsic error on the decoupling assessment, the baseline accuracy of cavity length measurement was first verified at 20 °C, where temperature interference is negligible.

**Figure 10:**
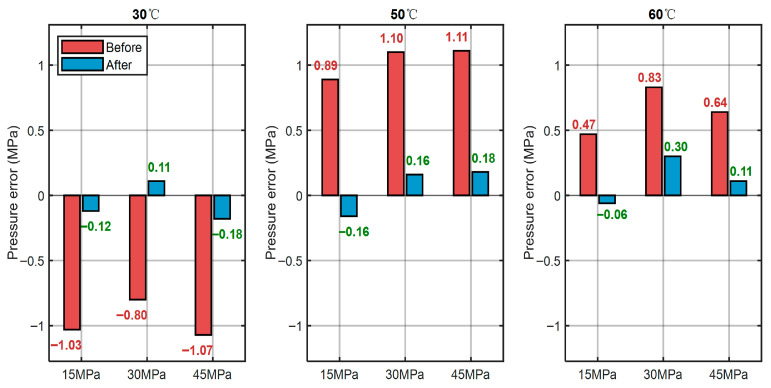
Comparison chart of measurement pressure and standard pressure errors before and after temperature-pressure decoupling at the same temperature but different pressures.

Table 2 lists the cavity lengths and corresponding pressure readings before and after applying the temperature–pressure decoupling algorithm. Figure 10 presents the pressure measurement results at different temperatures. Without compensation, temperature variations caused a clear drift in pressure readings, with errors reaching up to 1.11 MPa. Once the decoupling algorithm was applied, the residual errors at all tested pressure levels were reduced to within ±0.3 MPa, showing that the method successfully suppresses temperature-induced deviations.

**Figure 11:**
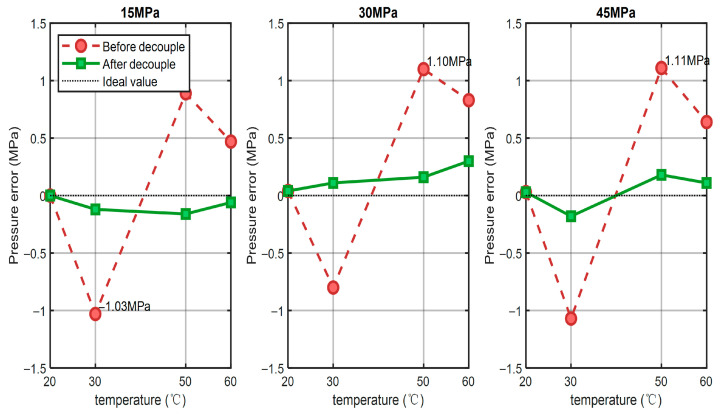
Line graph comparing maximum measurement errors at different temperatures for the same pressure with temperature-pressure decoupling.

**Figure 12:**
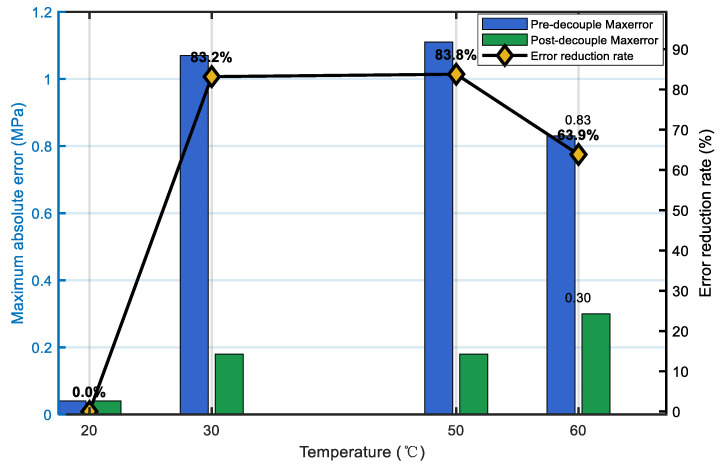
Performance statistics of the decoupling effect.

The influence of temperature drift at fixed pressure points is shown in Figure 11. At 15, 30, and 45 MPa, the uncompensated pressure readings consistently deviated with increasing temperature, with maximum errors above 1 MPa. In contrast, after compensation, the error curves remained close to zero across the entire temperature range, with fluctuations smaller than 0.3 MPa. This demonstrates that the algorithm effectively stabilizes the measurement against thermal variations. A more quantitative comparison is given in Figure 12. At 60 °C, for example, the maximum absolute error was reduced from 0.83 MPa to 0.30 MPa, corresponding to an error reduction of about 63.9%. Over the 30–60 °C range, the mean error reduction reached 83.4%, and the overall system accuracy improved to better than 0.2% FS.

Finally, combined pressure–temperature loading experiments were performed to verify practical performance. The uncompensated maximum error was about 0.3 MPa (0.5% FS). After applying the decoupling algorithm, the sensor achieved stable and accurate pressure readings across the full range, confirming its suitability for oil-well applications.

## 5. Discussion

The proposed FBG-assisted Fabry–Perot (FP) cavity pressure sensor shows clear advantages in suppressing temperature–pressure coupling under oil-well conditions.

First, regarding decoupling performance, the combined use of the FBG reference and the thermal expansion model effectively reduces temperature drift. For example, at 60 °C/45 MPa, the pressure error after decoupling was reduced to 0.18% FS (compared with 0.37 MPa without compensation), confirming that the method can maintain reliable accuracy under harsh conditions. The choice of Inconel 718 and SY-40 adhesive also contributes to structural stability at temperatures up to 100 °C, which is superior to conventional epoxy-based FP sensors that deform above 80 °C.

However, several sources of error remain. One is related to the fitting algorithm used for temperature compensation. In practice, we only measured integer temperature points between 20 and 100 °C, with 10 °C intervals. This limited dataset cannot fully capture the nonlinear temperature response of the FP cavity, as different materials in the sensor—metal housing, high-temperature adhesive, and fiber—exhibit different deformation behaviors at elevated temperatures. Consequently, the fitted curve cannot completely eliminate temperature-induced errors, which explains why residual errors are larger at certain high-temperature points. A second source of error arises from the pressure loading system: when the pressure pump maintains a set point, small fluctuations or jumps occur, and the recorded pressure values are not perfectly stable, which inevitably introduces measurement uncertainty [28].

In addition, practical deployment has shown that metals may exhibit hysteresis during elastic recovery, especially under rapid unloading. This can further increase errors during dynamic pressure changes. Sensor calibration itself also carries a small inherent uncertainty, and the quadratic term observed in the temperature sensitivity fitting suggests that thermal expansion is not purely linear. These factors together contribute to the remaining deviations observed in the experiments.

Looking ahead, further work will focus on improving the fitting accuracy, possibly by collecting denser temperature calibration data or by adopting more advanced compensation strategies. Data-driven methods such as machine learning may provide additional flexibility to model the nonlinearities, although such approaches require careful validation before deployment in oil wells.

Finally, the sensor addresses practical pain points in downhole monitoring, where conventional electronic sensors suffer from electromagnetic interference and thermal drift. With its high sensitivity (377 nm/MPa), compact size, and demonstrated decoupling capability, the proposed FP sensor shows strong potential for reliable pressure monitoring in narrow wellbores, which is critical for preventing blowouts and ensuring safe oil production.

## Figures and Tables

**Table 1:** Data on the length of the three-stage pressure chamber. For each test, the pressure was ramped up from 0 MPa to 60 MPa and ramped back down to 0 MPa.

Pressure	Cycle 1-Ramp Up	Cycle 1-Ramp Down	Cycle 2-Ramp Up	Cycle 2-Ramp Down	Cycle 3-Ramp Up	Cycle 3-Ramp Down
(MPa)	(nm)	(nm)	(nm)	(nm)	(nm)	(nm)
0	445,552.58	445,618.49	445,538.49	445,604.38	445,598.79	445,662.18
5	443,376.97	443,466.53	443,365.18	443,452.87	443,442.19	443,529.84
10	441,798.42	441,643.37	441,775.92	441,618.13	441,743.00	441,687.93
15	439,756.56	439,686.50	439,732.68	439,661.80	439,812.77	439,726.14
20	437,678.01	437,778.01	437,693.00	437,761.59	437,722.13	437,823.11
25	436,343.84	436,147.96	436,361.10	436,125.08	436,298.08	436,092.31
30	434,503.05	434,357.24	434,534.17	434,339.97	434,598.86	434,448.26
35	432,663.40	432,753.40	432,695.20	432,728.13	432,729.61	432,814.99
40	430,981.47	430,781.47	431,022.38	430,826.99	431,041.50	430,843.63
45	428,982.50	428,943.20	429,037.11	429,001.32	429,083.01	429,045.23
50	427,151.45	426,953.58	427,229.74	427,054.81	427,239.17	427,031.02
55	425,298.36	425,276.63	425,396.27	425,395.26	425,421.99	425,397.49
60	423,277.01	423,277.01	423,413.26	423,389.64	423,478.00	423,474.28

**Table 2:** Pressure measurement data table for sensors at different temperatures and pressures.

Temperature (°C)	Standard Pressure (MPa)	0 Pressure Room Temperature Cavity Length (nm)	Uncoupled Cavity Length (nm)	Decoupled Cavity Length (nm)	Test Pressure (Uncoupled) (MPa)	Test Pressure (Decoupled) (MPa)
20	15	445,517.25	439,853.07	439,853.07	15.00	15.00
20	30	445,517.25	434,175.83	434,175.83	30.04	30.04
20	45	445,517.25	428,521.41	428,521.41	45.03	45.03
30	15	445,517.25	440,241.26	439,901.95	13.97	14.88
30	30	445,517.25	434,493.29	434,153.98	29.20	30.11
30	45	445,517.25	428,933.01	428,593.70	43.93	44.82
50	15	445,517.25	439,517.88	438,814.67	15.89	14.84
50	30	445,517.25	433,781.08	433,073.87	31.10	30.16
50	45	445,517.25	428,110.64	427,407.43	46.11	45.18
60	15	445,517.25	439,676.50	438,948.69	15.47	14.94
60	30	445,517.25	433,880.34	433,152.53	30.83	30.30
60	45	445,517.25	428,291.56	427,563.75	45.64	45.11

## Data Availability

Dataset available on request from the authors.
